# Temporal interactions of plant - insect - predator after infection of bacterial pathogen on rice plants

**DOI:** 10.1038/srep26043

**Published:** 2016-05-17

**Authors:** Ze Sun, Zhuang Liu, Wen Zhou, Huanan Jin, Hao Liu, Aiming Zhou, Aijun Zhang, Man-Qun Wang

**Affiliations:** 1 Hubei Insect Resources Utilization and Sustainable Pest Management Key Laboratory, College of Plant Science and Technology, Huazhong Agricultural University, Wuhan 430070, P. R. China; 2 Invasive Insect Biocontrol and Behavior Laboratory, BARC-West, USDA-ARS, Beltsville, MD 20705-2350, USA.

## Abstract

Pathogenic infection on plants may affect interactions of host-plants with their herbivores, as well as the herbivores with their predators. In this study, the effects of infection by pathogenic bacterium *Xanthomonas oryzae* pv. *oryzae* (*Xoo*), which causes a vascular disease in rice, on rice plants and consequent interactions with a rice herbivore, brown rice planthopper (BPH) *Nilaparvata lugens*, and its major predator, *Cyrtorhinus lividipennis*, were investigated. The results showed that the rice plants exhibited increased resistance to BPH only at 3 d post-inoculation of *Xoo*, while the *Xoo* infection did not affect the development and fecundity of BPH. BPH exhibited a higher preference to *Xoo* infected rice plants, whereas *C. lividipennis* preferred the *Xoo* infected rice plants after BPH fed, but preferred healthy rice plants without BPH fed. Volatile organic compounds emitted from *Xoo* rice were significantly higher than those from healthy rice plants, *Xoo* infection on BPH fed plants caused rice plants to emit more the herbivore-induced plant volatiles, while all of these changes correlated to the temporal dimension. These results demonstrated that *Xoo* infection significantly influenced the interactions of rice plants with two non-vectors, BPH and its predator, although these effects exhibited in a temporal pattern after infection.

In nature, plants are attacked by a multitude of predators, including herbivorous arthropods and plant pathogens^1^,^2^. In response, plants have evolved defense strategies that enable them to recognize herbivores or pathogens^3^ and, as a consequence, in many cases, reduced the extent of resulting damage^4^.

Several recent studies have shown that complicated interactions exist between pathogens and herbivores that feed on the same plant, in addition to their independent effects on the host plant^5^. A range of physical or chemical defense responses can affect the performance of herbivores, and even impact the herbivore community^6^,^7^,^8^,^9^. In addition, pathogen infection alters the emission of volatile organic compounds (VOCs) or visual cues, both of which have important roles in mediating ecological interactions among plants and insects^10^,^11^,^12^,^13^. The VOCs are diverse compounds and are also known to have a strong ecological significance^14^. Herbivore species respond differently to infection of plant pathogens; thus, pathogen attack can result in complicated effects on insect communities of the host plant^9^,^15^,^16^. For example, endophagous insects exhibited different preferences for the infected and uninfected parts of the creeping thistle *Cirsium arvense* when treated with a rust fungus *Puccinia punctiformis*, although this treatment had no effect on ectophagous insects^17^. Furthermore, the herbivore-induced plant volatiles (HIPVs), which are considered to be an important ecology function of attracting the herbivores’ natural enemies^16^,^18^, and could be affected by plant pathogens infection. Some research reported that the change of HIPVs caused by pathogens infection did not affect the preference of nature enemy^19^, while it has been reported higher rates of parasitism of aphids were found on the endophytic fungi infect Italian ryegrass *Lolium multiflorum*^20^.

Similarly, damage inflicted by herbivorous insects induces chemical and physiological changes in plants, which affects the pathogen infection. Physiological changes in cotton seedlings caused by previous exposure to spider mites, *Tetranychus urticae*, reduced the probability of infection and severity of the symptoms caused by the wilt fungus *Verticillium dahliae*^15^. Feeding by the white-backed planthopper, *Sogatella furcifera*, induced resistance to rice blast caused by *Magnaporthe grisea*^21^,^22^,^23^. In contrast, the susceptibility of willow towards infection by the rust *Melampsora allii-fragilis* was increased by feeding by the leaf willow beetle, *Plagiodera versicolora*, as indicated by a higher number of rust sori on leaves adjacent to feeding-damaged leaves^24^. Furthermore, pre-infestation by *S. furcifera* conferred resistance to bacterial blight caused by *Xanthomonas oryzae* pv. *oryzae* (*Xoo*) in rice plant, while infestation by the brown planthopper (BPH) *Nilaparvata lugens* (Stål), did not significantly reduce the incidence of bacterial blight symptoms^25^.

Furthermore, the interaction between a pathogen and a herbivore depends on the timescale, given that the systemic acquired resistance induced by pathogens needs time to develop and recede^26^,^27^. A temporal scale experiment showed that *P. versicolora* avoided feeding on leaf tissue of the willow hybrid *Salix x cuspidate* with rust fungus *M. allii-fragilis* infection; infected leaves were avoided at all the times tested (at 8, 12, and 16 days after infection), whereas symptom-free leaves were only avoided at 16 days after infection^28^. Research of the impacts of pathogen attacking of plant on herbivore communities on the temporal scale can help us to gain insight into the interactions between pathogens and herbivores and, therefore, is useful for protecting plants of economic value from herbivorous pests^9^,^29^.

In this study we investigated the temporal change of preferences of BPH and its major predator *Cyrtorhinus lividipennis* Reuter and BPH performance (development and reproduction) in rice plants after *Xoo* infection at different stages of infection (1–5 d, 10 d and 15 d). We also examined whether *Xoo* infection altered plant-derived chemicals that mediate interactions between the host plants, BPH, and their predator, and whether *Xoo* infection influenced the host location behavior of non-vector BPH and their predator. Furthermore, Electrical Penetration Graph (EPG) technology was applied to investigate the temporal change of the resistance level of rice to BPH after *Xoo* infection and to explore the relationship between these complicated plant-based ecological communities.

## Results

### BPH feeding behavior

**Figure 1:**
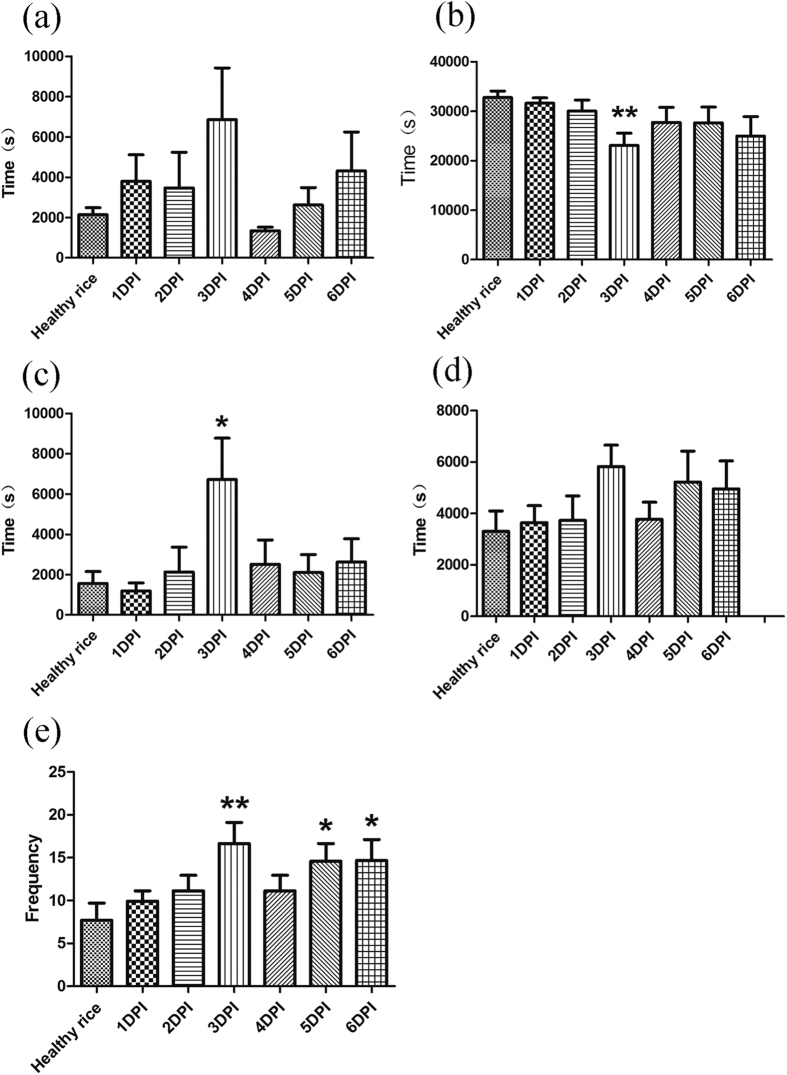
BPH activities as identified by electrical penetration graph (EPG) recordings within the first 12 h at 1 to 6 days after *Xoo* infestation (mean ± SE, n = 9–15). PI: plants infected by *Xoo*. (**a**) Time interval to the 1st N4a. (**b**) Total duration of N4b. (**c**) Total duration of np. (**d**) Total duration of ph. (**e**) Total number of ph. Waveform: np (non-penetration), ph (path way waveform), N4a (Sieve element salivation), N4b (ingestion in phloem). Asterisks show significant differences from the control group (*p < 0.05; **p < 0.01).

**Figure 2:**
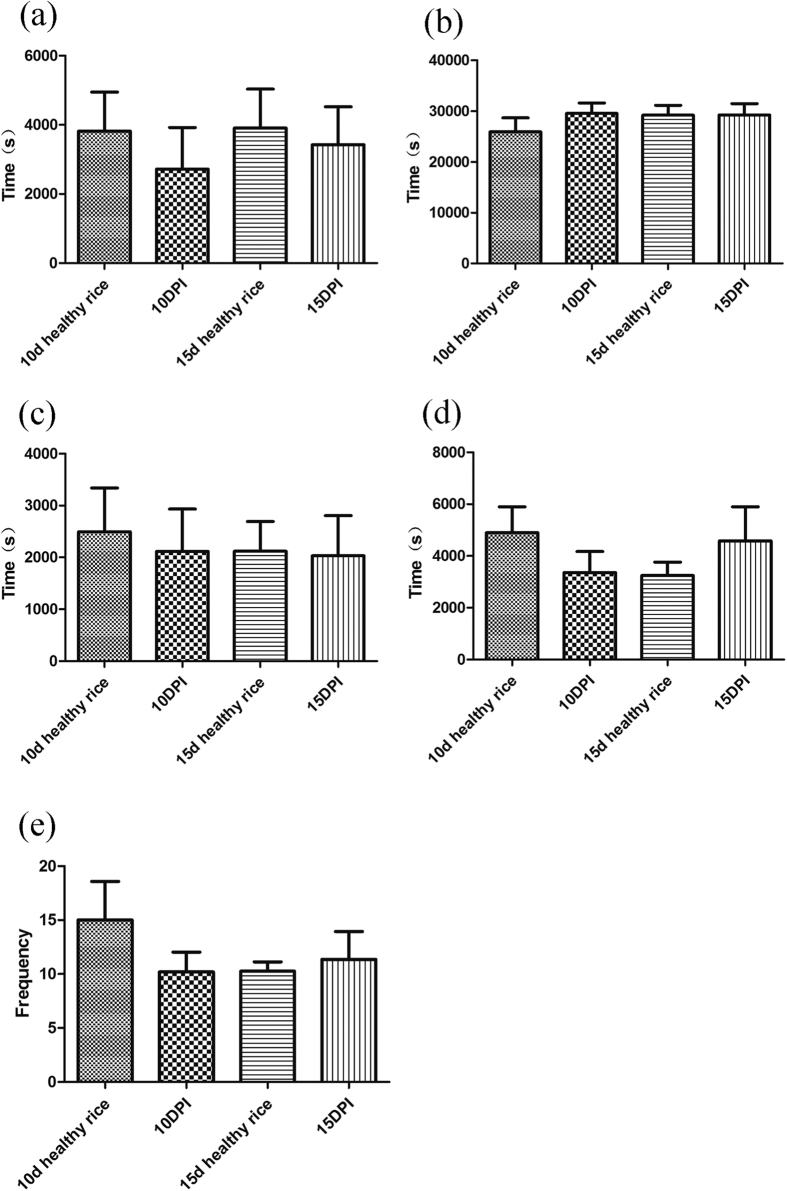
BPH activities as identified by electrical penetration graph (EPG) recordings within the first 12 h at 10 and 15 days after *Xoo* infestation (mean ± SE, n = 9–15). PI: plants infected by *Xoo*. (**a**) Time interval to 1st N4a. (**b**) Total duration of N4b. (**c**) Total duration of np. (**d**) Total duration of ph. (**e**) Total number of ph. Waveform: np (non-penetration), ph (path way waveform), N4a (sieve element salivation), N4b (ingestion in phloem).

The EPG analysis showed that, at 3 d after inoculation, there were significant differences in the parameters of BPH feeding behavior between *Xoo* treatment and control healthy groups. The total duration of np waveform was significantly longer than on healthy rice (*t *= 2.42, *p *= 0.031) (Fig. 1c) and the time interval to the first N4a waveform appear was 3.2 times longer than that on healthy rice (Fig. 1a). In addition, the total number of ph waveforms of BPH on infected rice plants was significantly more than that on healthy rice plants (*t *= 2.48, *p *= 0.009) (Fig. 1e), whereas the total duration of N4b waveforms of BPH on infected rice plant was less than that on healthy rice plant (*t *= 3.52, *p *= 0.002) (Fig. 1b). These differences indicated that BPH took more efforts to puncture the rice plants surface during the feeding process, and fed for less time on phloem from infected rice plants at 3 d post-inoculation. In contrast, except the total number of ph waveforms of BPH on 5 d post-inoculation (*t *= 2.37, *p *= 0.027) and 6 d post-inoculation (*t *= 2.21, *p *= 0.039) (Fig. 1e), most of other the parameters of BPH feeding on infected rice plants in other days post-inoculation were similar to those of BPH on healthy rice plants (Figs 1 and 2; Table S1).

### Fecundity and development of BPH

**Figure 3:**
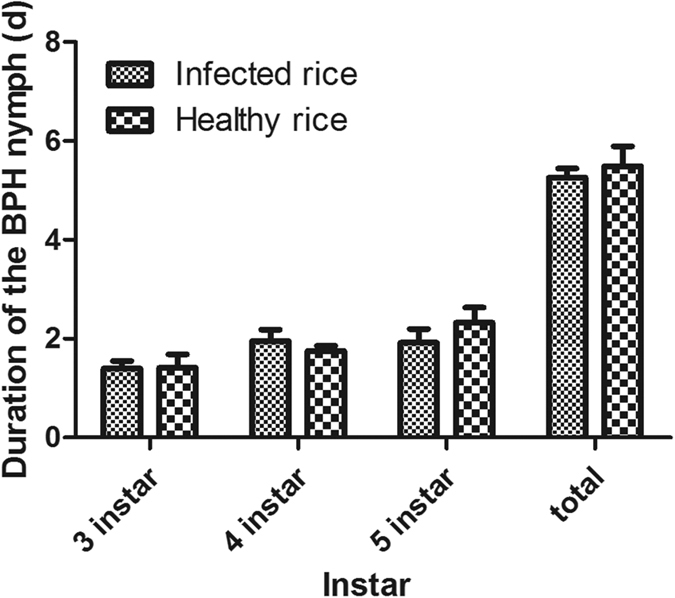
The duration (mean ± SE, n = 5) of BPH nymph of 3th instar, 4th instar, 5th instar and the total duration from 3th instar to newly emerged adult on healthy and infected rice plants. PI: plants infected by *Xoo* for 5 days.

The data showed that *Xoo* infection had no significant effect on the performance of BPH. The fecundity and hatchability of BPH on infected rice plants were similar to those of BPH on healthy rice plants (Table 1). In addition, the duration of 3–5 instar and the total duration from 3th instar to newly emerged adult BPH were similar between infected and healthy rice plants (Fig. 3).

### Herbivore behavior

**Figure 4:**
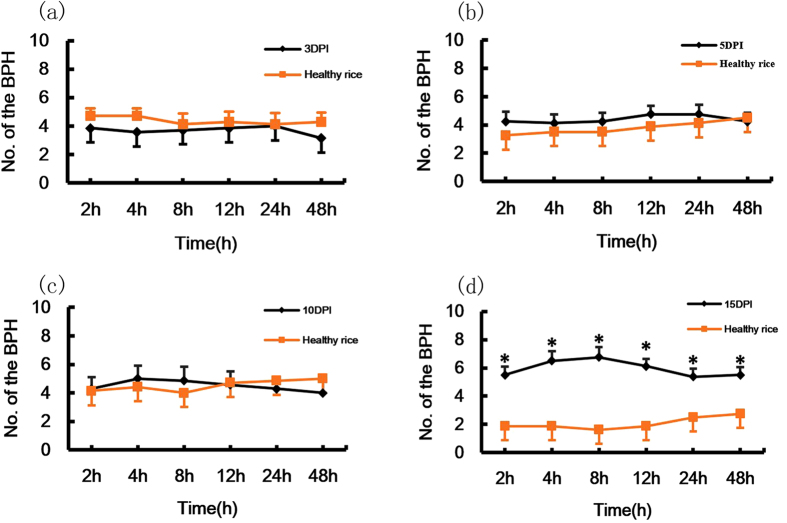
The number (mean ± SE, n = 7–8) of female BPH adults in two-choice assays between healthy rice plants and rice infected by *Xoo* in semi-natural foraging experiments. PI: plants infected by *Xoo*. (**a–d**) the number of the BPH on plants at 3, 5, 10, 15 days after treatment with (dark line) or without (orange line) *Xoo*. Asterisks show significant differences among the treatments (*p < 0.05).

**Figure 5:**
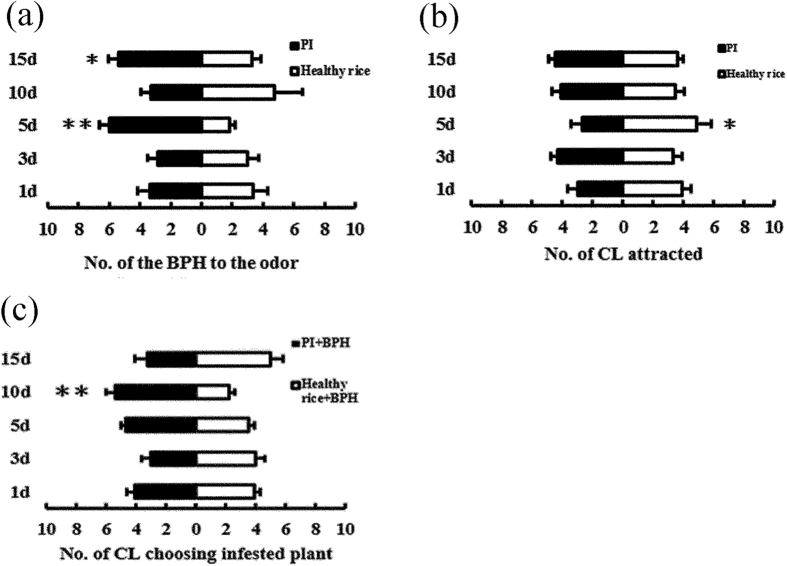
The number of BPH and *C. lividipennis* attracted by volatiles released from the different rice treatments. (**a**) The number (mean ± SE, n = 6–10) of macropterous BPH female and male adults in H-tube tests between healthy rice plants and rice plants infected by *Xoo* at different days. (**b**) The number (mean ± SE, n = 7–9) of *C. lividipennis* adults in H-tube tests between healthy rice plants and rice plants infected by *Xoo* at different days. (**c**) The number (mean ± SE, n = 6–8) of *C. lividipennis* adults in H-tube tests between healthy rice plants and rice plants infected by *Xoo* after fed by BPH. PI: plants infected by *Xoo*. CL: *C. lividipennis*. Asterisks show significant differences among the treatments (*p < 0.05; **p < 0.01).

Two-choice tests were performed to assess the host preference and location behavior of BPH in response to *Xoo*-infected and healthy rice plants. The results of semi-natural foraging experiments showed the number of BPH presented on infected rice plants at 15 d post-inoculation were 2–4 times more than that on healthy rice plants (2 h, *t* = 3.34, *p *= 0.012; 4 h, *t *= 3.97, *p *= 0.005; 8 h, *t *= 4.26, *p *= 0.004; 12 h, *t *= 4.82, *p *= 0.002; 24 h, *t *= 2.59, *p *= 0.036; 48 h, *t *= 2.54, *p *= 0.038), while there were no significant differences between infected and healthy rice plants at other time points (Fig. 4). The results of the H-tube choice experiment revealed that adult female BPH exhibited preferences to infected rice plants at 5 d (*X*^*2*^* *= 24.60, *p* < 0.0001) and at 15 d (*X*^*2*^* *= 5.07, *p *= 0.024) post-inoculations, whereas there were no significant differences at the other time points (Fig. 5a).

### Predator behavior

Without BPH feeding, *C. lividipennis* adults exhibited a significantly higher preference to the healthy rice plants at 5 d post-inoculation (*X*^*2*^* *= 4.25, *p *= 0.039), whereas at other time points no significant differences were observed between infected rice and healthy rice plants (Fig. 5b). In contrast, after rice plants were fed by with BPH, *C. lividipennis* adults showed a higher preference for *Xoo* infected rice at 10 d post-inoculation (*X*^*2*^* *= 12.19, *p *= 0.0005) and there were no significant differences detected between infected rice and healthy rice at other time points (Fig. 5c).

### Analysis of VOCs

**Figure 6:**
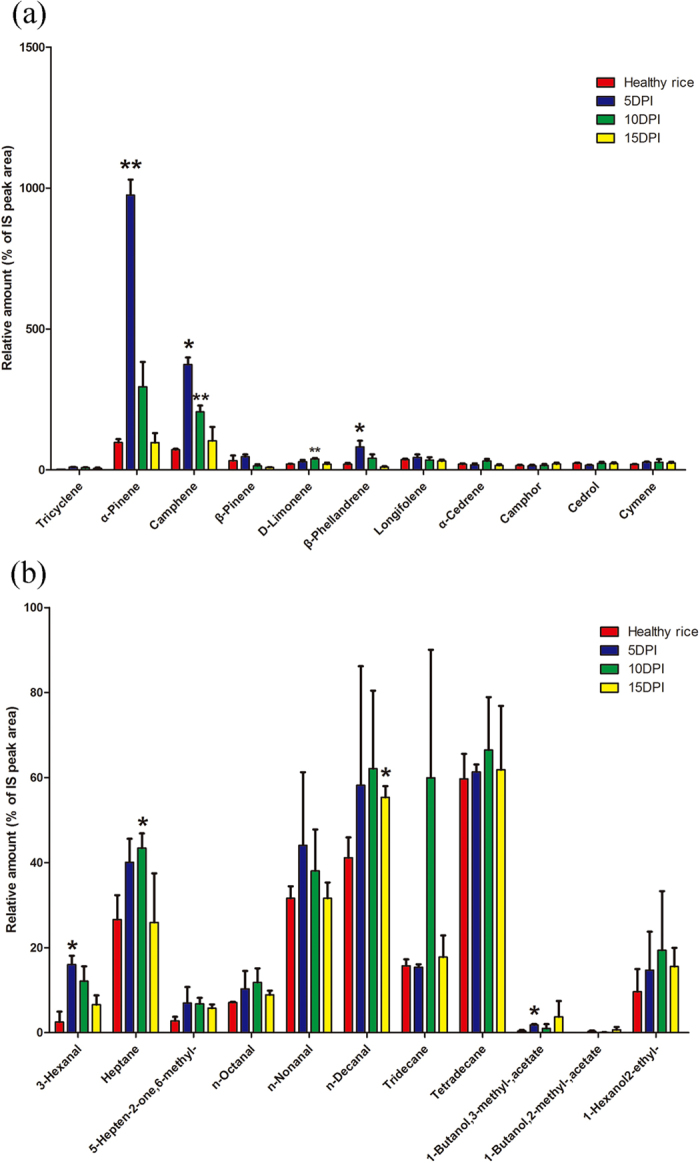
The amounts of volatile compounds (% of internal standard + SEM, n = 3–4) emitted from health rice and rice infected by *Xoo* at 5 d,10 d and 15 d. PI: plants infected by *Xoo*. (**a**) Terpenes. (**b**) Aldehyde, ketone and esters. Asterisks show significant differences among the treatments (*p < 0.05; **p < 0.01).

**Figure 7:**
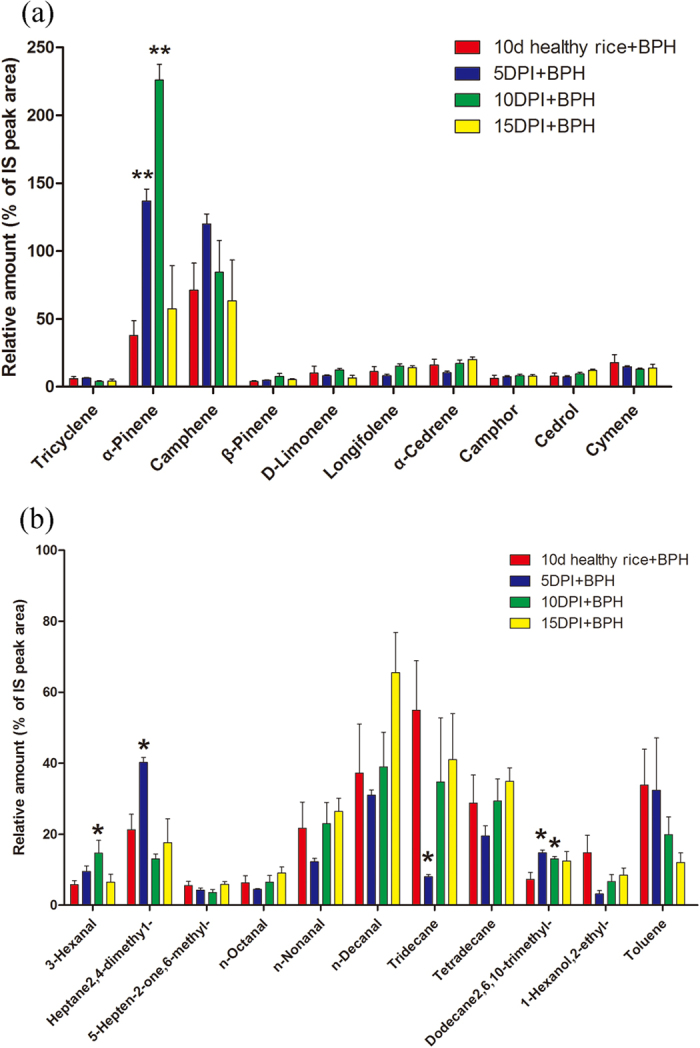
The amounts of volatile compounds (% of IS peak area + SEM, n = 3–5)emitted from healthy rice induced by BPH feed and rice infected by *Xoo* at 5 d,10 d and 15 d, meanwhile, have suffer from BPH feed for 24 h. PI: plants infected by *Xoo*. (**a**) Terpenes. (**b**) Aldehyde, ketone and esters. Asterisks show significant differences among the treatments (*p < 0.05; **p < 0.01). Some materials didn’t appear in this figure, because they were too few to detect in these sample.

More than 70 VOCs were detected with GC/MS (Table S2) and among these VOCs, terpenes, aldehydes, ketones, and esters which play a significant role in affecting the behavior of BPH and *C. lividipennis*^18^ and some high amount and highly volatile hydrocarbons were shown in our figure. In our study, without BPH feeding, the amount of α-Pinene (*t *= 20.00, *p *= 0.0003), Camphene (*t *= 12.04, *p *= 0.049), β-Phellandrene (*t *= 3.5, *p *= 0.039), 3-Hexanal (*t *= 3.79, *p *= 0.032) and 1-Butanol,3-methyl-,acetate (*t *= 4.16, *p *= 0.025) from *Xoo*-infected rice at 5 d post-inoculation, and amount of D-Limonene (*t *= 4.86, *p *= 0.005), Camphene (*t *= 6.05, *p *= 0.008) and Heptane (*t *= 2.67, *p *= 0.044) at 10 d post-inoculation were significantly higher than those from healthy rice plants, and the amount of Camphene is only half than those from *Xoo*-infected rice at 5 d post-inoculation. However, except n-Decanal (*t *= 2.79, *p *= 0.038) the amounts of all VOCs from *Xoo*-infected rice at 15 d post-inoculation didn’t have significantly difference to those of healthy rice plants (Fig. 6). In contrast, with BPH infection, the amounts of three hydrocarbons, 2,4-Dmethyl-heptane (*t *= 3.20, *p *= 0.018), 2,6,10-Trimethyl-dodecane (*t *= 2.78, *p *= 0.032) and α-Pinene (*t *= 6.29, *p *= 0.0007) from *Xoo*-infected rice at 5 d post-inoculation were significantly higher than those from healthy rice and the amounts of Tridecane (*t *= 3.37, *p *= 0.028) significantly lower than those from healthy rice; Higher amount of α-Pinene (*t *= 11.25, *p* < 0.0001), 3-Hexanal (*t *= 2.35, *p *= 0.047) and 2,6,10-Trimethyl-dodecane (*t *= 2.75, *p *= 0.025) from *Xoo*-infected rice were detected at 10 d post-inoculation and the amount of α-Pinene was about 1.6 times more than those from *Xoo*-infected rice at 5 d post-inoculation (Fig. 7).

## Discussion

Many studies have shown that the pathogen infection can affect the performance and preference of herbivorous insects^10^,^30^,^31^, and that this influence, although variable, can have a systemic effect beyond the infected site^9^,^24^,^32^. In this study, we investigated whether the infection of pathogen *Xoo* could affect the performance and preference of BPH as well as the preference of its predator *C. lividipennis*, at 1 d, 3 d, 5 d, 10 d, 15 d post-infection.

In our study, the fecundity and developmental duration of BPH showed no obvious change on *Xoo*-infected rice, but the EPG data showed that *Xoo* infection affected the feeding behavior of BPH on rice plant at 3 d post-infection. BPH feeding can be divided into two main categories based on the EPG waveforms^33^. The first category, revealed by the np, ph, and N5 waveforms, represents the phase of BPH finding the feeding site to reach the sieve element, and the second category represents ingestion activities in the sieve element, including N4a and N4b waveforms^33^,^34^,^35^. Therefore, the longer first phase and the shorter second phase showed that BPH spent more time beginning ingestion and less time ingesting phloem on *Xoo*-infected rice 3 d post-inoculation, indicating an obvious resistance in *Xoo*-infected rice 3 d post-inoculation. It has been suggested that constitutive chemicals and inducible defenses are activated by pathogen infection, including the production of secondary metabolites and structural changes in the plant tissue^36^,^37^ and that these defense mechanisms increase the resistance of the plant to herbivore attack, affecting the feeding capacity of the insect^30^, but these obvious differences were only detected at 3 d post-inoculation in this study supports the classical theory that pathogen infection induces the systemic acquired resistance of a plant, which varies with the time since inoculation and recedes after a certain period^26^. This also explains that the fecundity and developmental duration of BPH showed no obvious change on *Xoo*-infected rice at 3 d after inoculation since systemic acquired resistance have already receded. Several studies have shown that pathogen infection of plants had impacts on the performance of herbivore, while the impact of host plant pathogens on different insect species ranged from negative to positive^30^,^32^,^37^,^38^,^39^,^40^. Tack *et al*. reported that insect species reacted differently to the presence of the same plant pathogen, *Erysiphe alphitoides*, which caused powdery mildew in oak, *Quercus robur*. Fungal infection reduced the growth rate, growth efficiency, and pupal mass of the free-feeding caterpillar, *Acronicta psi*. In contrast, it increased the growth rate, decreased the developmental time, and increased the parasitism rate of the leaf miner, *Tischeria ekebladella*^30^. Infection of single leaflets of tomato by *Pseudomonas syringae* pv. *tomato* have no effect on *Helicoverpa zea* larva^40^.

Our results also showed that the preference of BPH was affected in a temporal pattern after *Xoo* infection. BPH exhibited a higher preference for *Xoo*-infected rice plants only at 5 d after and 15 d post-inoculations in the H-tube choice experiments. To further reveal the mechanism behind this preference change, we analyzed the amount of VOCs released after *Xoo* infection, given that VOCs are the key for understanding the preference change of herbivores. Our results indicated that the amounts of some VOCs, including terpenes and green leaf volatile play a significant role in affecting the behavior of BPH^18^, changed significantly on a temporal scale. Amounts of terpenes and green leaf volatiles in pathogenic-infected rice plants were significantly increased at 5 d post-inoculation and then decreased to the levels of healthy rice plants. It seems that initially high amount of VOCs attracts BPH and after that, the proportion of different VOCs plays an important role in attracting BPH^41^,^42^,^43^,^44^. However, the function of the amount and proportion of VOCs in terms of attracting non-vector BPH requires further investigation. These results also prove the fact that pathogen affection alters plant VOCs emissions and as a consequence this alteration affects the interactions between the host plant and its herbivores. These changes are known to influence the olfactory responses of insects by either inhibiting or enhancing their attraction and/or oviposition^45^,^46^,^47^,^48^. It has been suggested that avoidance of oviposition by herbivorous insects on leaves infected by a fungal pathogen is an adaptive strategy to aid the fitness of the offspring^49^. Disease-induced plant VOCs were repellent to aphids, and 2-pentadecanone was the key semiochemical underpinning the repellent effect^31^. Furthermore, our results indicated that the preference of insect varies after pathogen infection on the time-scale. This preference variation may ensure the dispersal of pathogens to other host plants via the attraction of insect vectors^50^,^51^,^52^,^53^,^54^, or decreasing the attraction of non-vectoring insects^55^.

Furthermore we observed that in the semi-natural foraging experiment, the BPH showed a preference only for *Xoo*-infected rice plants at 15 d post-inoculation. This result suggests that not only plant VOCs, but also other cues may be involved in host location by BPH, such as visual cues, either alone or in combination with VOCs. Ponzio *et al*.^4^ suggested that, in wasps, VOC-mediated foraging should be labile when the VOC signal was not a reliable indicator of the attacking species or when the wasp was not able to distinguish subtle differences in VOC blends^4^. The VOCs emitted from plants induced by non-hosts could be attractive to *Cotesia glomerata* wasps; for example, infection with the cucumber mosaic virus alters the host-plant phenotype both visually and chemically and these changes influence plant interactions with vector and non-vector insects^13^.

Previous studies have shown that the plant pathogenic attacking affects the HIPVs release. The herbivore-fed maize plants at 72 h post-infection with a necrotrophic fungus *Setosphaeria turcica* emitted 47% less volatiles than the plants without fungus infection^19^, but *Spodoptera littoralis*’ natural enemy *Cotesia marginiventris* and *Microplitis rufiventris* was still able to locate their host when infected with fungus and responded equally to healthy and infected plants at 72 h post-infection^19^. Our study indicated that *Xoo* infection affects *C. lividipennis* preference and that the effects of plant–pathogen–insect interactions vary on the time-scale after the infection of pathogen *Xoo* reveled by two behavioural experiments (*C. lividipennis* preference after *Xoo* infection with BPH feeding treatment or not). First, without BPH feeding, *C. lividipennis* showed avoidance behavior in response to rice plant at 5 d post-inoculation. This avoidance behavior can be associated with the high amount of VOCs released from *Xoo*-infected plants at 5 d post-inoculation, suggesting that the amount of VOCs released would affect the behavior of natural enemy even if the absent of pest harm. In addition, the other behavioral experiments indicated that after BPH feeding, *C. lividipennis* prefered the rice plants at 10 d post-inoculation, although the *C. lividipennis* responded equally at other time points examined. This result is consistent with that plants released significantly more HIPVs at 5 d and 10 d after *Xoo* infections; and at 10 d post-infection, the amount of terpenes (α-Pinene) and green leaf volatiles (3-Hexenal) reached the highest amount.

In summary, our study established the fact that the *Xoo* infection had an obvious effect on the feeding behavior, but not the performance of BPH, and the variation of VOCs and HIPVs after *Xoo* infection lead to the changes of the preferences of BPH and of its natural enemy *C. lividipennis* on the temporal dimension. Our results provided insight into the temporal variability of interactions between plants, pathogen, and insects, as well as the information of plant resistance to pests and diseases, and given more cues to help us to know the role of odor which rice release to used for *C. lividipennis* and BPH to find the host, which will contributed to understanding of plant-based communities.

## Methods

### Plant and insect cultures, and bacterial inoculations

The rice variety used in this study was Minghui 63 (MH63), a restorer line of Indica rice (*Oryza sativa* L.). The pre-germinated seeds were sown in clay pots [10 cm (diam) × 8 cm (height)] in a greenhouse under natural light conditions at 28 ± 4 °C and with 70–80% relative humidity (RH). Each pot contained one seed supplied with 0.5 g compound fertilizer (N:P:K = 14%:16%:15%) and was watered daily. Sixty-day-old plants were used for all experiments.

BPH and *C. lividipennis* were collected from rice fields in Wuhan, China. They were continuously reared on the TN1 rice variety (a variety susceptible to BPH) in net cages in the same greenhouse.

*Xoo* strain XG-25 (race 4), which is virulent to MH63, was used in this study. The colonies were maintained in potato-sucrose-agar medium at 4 °C for routine work. A single colony was inoculated in lysogeny broth liquid medium and grown at 220 rpm for 10 h at 37 °C. The bacterial suspension was then normalized to a concentration of approximately 10^8^ colony-forming units (cfu) ml^−1^ in sterilized distilled water, and used for rice inoculation.

**Figure 8:**
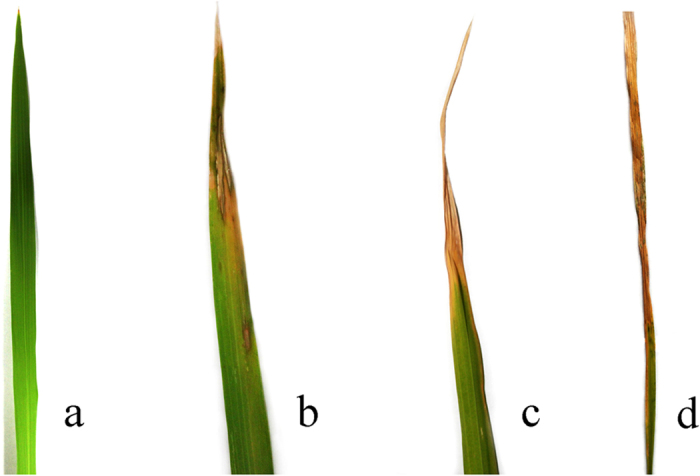
Disease symptoms of bacterial blight. (**a**) The control rice flag leaf. (**b–d**) Disease symptoms of bacterial blight at 5, 10, 15 days post inoculation with *Xanthomonas oryzae* pv. *oryzae* (*Xoo*), respectively.

The rice plants were inoculated by spraying the bacterial suspension until runoff and the control rice plants were treated with sterile lysogeny broth liquid medium. After inoculation, the rice plants were maintained in a cabinet (30 ± 1 °C, RH = 90 ± 5%, 20000 lx light intensity) for 12 h, and then transferred to a greenhouse and grown under the same conditions as previously described. *Xoo*-infected and healthy rice plants at 1, 3, 5, 10, and 15 days after inoculation were used for data collection, and obvious disease symptoms will be visible on the rice plants about at 4–5 days after *Xoo*-infection (Fig. 8). To make sure plants with the same infected level, the rice plants which have almost same disease symptoms were used for herbivore performance (5 d post *Xoo*-infected rice plants) and VOCs collection (5 d, 10 d, 15 d post *Xoo*-infected rice plants).

### Feeding behavior of BPH

Rice plants at 1–6, 10, and 15 days after inoculation of *Xoo* were used in this experiment. The feeding behavior of macropterous adult female BPH was recorded using a Giga-4 DC EPG equipment (Wageningen Agricultural University, Wageningen, Netherlands). A 3-cm long and 18.5-mm diameter gold wire was connected to the dorsum of the insects with conductive silver glue; the wired insects were then carefully placed onto the stem of the rice plants. The gain of the amplifier was 50× and the plant voltage was set to an output voltage of ±5 Vs. The EPG waveform of BPH performance in rice plants was recorded with Style+b software (Wageningen, Netherlands) for 13 h in a Faraday cage, and the waveforms from the first 12 h were used for data analysis.

All EPG recordings were conducted at 25 ± 2 °C, 60 ± 10% RH, and 12 variables were used to analyze the feeding behavior of BPH. Data represented the average of 9–15 replicates. EPG waveforms were identified and categorized according to the method described by Seo *et al*.^34^ and Ghaffar *et al*.^34^,^35^. Based on their occurrence sequences, a series of parameters representing different biological information were selected to reveal the impact of *Xoo* infection on BPH feeding behavior^56^.

### Herbivore performance

To test whether BPH developing performance on the rice plants was influenced by *Xoo* infection, the development and fecundity of BPH on healthy and infected rice plants were determined. Rice plants at 5 days after inoculation of *Xoo* were used for the following experiments. Ten newly emerged third-instar BPH nymphs were released into a plastic tube (2 cm in diameter, 10 cm in height) containing a single rice pot. The BPH nymphal instars were recorded daily until all BPH nymphs had emerged. In another experiment, two pairs of newly emerged brachypterous female and male adults were released into a single rice pot covered with a plastic tube (13 cm in diameter, 50 cm in height), and the adult BPH were removed after 10 days. The number of hatched nymphs and unhatched eggs resulting from each pair of BPH were recorded daily. The data collected were the average of 5–6 replicates.

### Rice plant preferences of BPH and *C. lividipennis*

In semi-natural foraging test, ten gravid adult macropterous BPH females were introduced into a cage (40 cm × 40 cm × 70 cm) containing four plants (two infected rice plants and two healthy rice plants) and the number of BPH on each rice plant was determined at 1, 2, 4, 8, 24, and 48 h after their introduction.

To further confirm the previous results, in a parallel experiment, a sealed system was set up comprising two plastic tubes (13 cm in diameter, 60 cm in height) individually containing one of a pair of two plants (an infected rice plant and a healthy rice plant). Two H-tubes similar to those described by Khan *et al*.^57^ were attached to each of the plastic tubes. These two H-tubes were connected by another tube (5 cm in diameter, 20 cm long, with a hole of 1 cm in diameter in the middle to release the insects). Ten female and ten male macropterous adult BPH were introduced into the H-tube. The number of BPH in each tube was counted at 30 min after their introduction.

The impact of *Xoo* infection on the preference of *C. lividipennis*, a predatory natural enemy of BPH, to rice plants was tested by H-tube choice assays^58^. After each rice plant was infested with 15 gravid female adults for 24 h, BPH were removed before experiment, then ten *C. lividipennis* adults were introduced into the H-tube for each test, and the number of *C. lividipennis* in each tube was counted 10 min later. Corresponding control experiments of rice plants without BPH feeding were performed to investigate whether the preference of *C. lividipennis* was affected only by the VOCs change. Each experiment was repeated 6–10 times.

### Collection and analysis of plant VOCs

Plant VOCs were collected with a closed loop dynamic headspace sampling system, similar to the method described by Sun *et al*.^59^. One milliliter of n-Hexane (Tedia, USA) was applied to elute VOCs from absorbent traps and 1300 ng Nonyl acetate (Sigma, Switzerland) was added to each sample as an internal standard. VOCs emitted from the *Xoo* infected rice and control rice plants were collected for 24 h (16 h in light and 8 h in darkness) at room temperature; BPH-induced plant volatiles from rice plants (each rice plant was also infested with 15 gravid female adults for 24 h) were collected for 8 h in the light (20000 lx) as described by Lou *et al*.^60^. Each treatment contained three to five biological replicates.

Gas chromatography–mass spectrometry (GC/MS) analyses of VOCs were performed on a QP-2010 GC/MS instrument (Shimadzu, Japan) equipped with an HP-5 MS fused-silica column (30 m × 0.25 mm × 0.25 μm). (Agilent Technologies, http://www.agilent.com). Helium (1 ml/min) was used as the carrier gas, and the initial oven temperature was 40 °C, held for 1 min, ramped at 8 °C min^−1^ to 300 °C held for 5 min. VOCs were identified by comparing their GC retention indices and MS spectra with those from the NIST11 library. The Retention index for each compound was determined using a series of straight chain alkanes (C7-C30) as standards.

### Data analysis

The EPG data, BPH performance, plant VOCs, and the foraging behavior in semi-natural experiment were analyzed by Student’s *t*-test to detect the difference between each treatment of *Xoo* infected rice and healthy rice. Chi-squared tests were used to evaluate the differences between H-tube behavioral responses of the BPH and *C. lividipenni*.

## Additional Information

**How to cite this article**: Sun, Z. *et al*. Temporal interactions of plant - insect - predator after infection of bacterial pathogen on rice plants. *Sci. Rep*. **6**, 26043; doi: 10.1038/srep26043 (2016).

## Supplementary Material

Supplementary Information

## Figures and Tables

**Table 1:** Fecundity and hatchability of BPH on infected rice and control rice plants.

	Rice plants infected by *Xoo*	Healthy rice plants
Number. of the Egg	474.94 ± 33.87	440.61 ± 39.35
Hatching rate (%)	63.62 ± 10.75	63.89 ± 7.66

Note: The number of BPH eggs (mean ± SE, n = 6, 2 female and 2 male BPH adults oviposited for 10 days). Hatching rate of BPH (mean ± SE, n = 6).

## References

[b1] Strong D. R., Lawton J. H. & Southwood S. R. Insects on plants. Community patterns and mechanisms. (Blackwell Scientific Publicatons, 1984).

[b2] Agrios G. Plant pathology. (Elsevier Academic Press, 2004).

[b3] Mithöfer A. & Boland W. Recognition of herbivory-associated molecular patterns. Plant Physiol. 146, 825–831 (2008). 18316636 10.1104/pp.107.113118 PMC2259064

[b4] Ponzio C., Gols R., Pieterse C. M. & Dicke M. Ecological and phytohormonal aspects of plant volatile emission in response to single and dual infestations with herbivores and phytopathogens. Funct. Ecol. 27, 587–598 (2013).

[b5] Pieterse C. M., Van der Does D., Zamioudis C., Leon-Reyes A. & Van Wees S. C. Hormonal modulation of plant immunity. Annu. Rev. Cell Dev. Biol. 28, 489–521 (2012). 22559264 10.1146/annurev-cellbio-092910-154055

[b6] Walters D. Plant defense: warding off attack by pathogens, pests and vertebrate herbivores. (Wiley-Blackwell, 2011).

[b7] Iason G. R., Dicke M. & Hartley S. E. The ecology of plant secondary metabolites: from genes to global processes. (Cambridge University Press, 2012).

[b8] McMenemy L. S., Hartley S. E., MacFarlane S. A., Karley A. J., Shepherd T. & Johnson S. N. Raspberry viruses manipulate the behaviour of their insect vectors. Entomol. Exp. Appl. 144, 56–68 (2012).

[b9] Tack A. J. & Dicke M. Plant pathogens structure arthropod communities across multiple spatial and temporal scales. Funct. Ecol. 27, 633–645 (2013).

[b10] Shapiro L., Moraes C. M., Stephenson A. G. & Mescher M. C. Pathogen effects on vegetative and floral odours mediate vector attraction and host exposure in a complex pathosystem. Ecol. Lett. 15, 1430–1438 (2012). 22988893 10.1111/ele.12001

[b11] Alexander H., Mauck K., Whitfield A., Garrett K. & Malmstrom C. Plant-virus interactions and the agro-ecological interface. Eur. J. Plant Pathol. 138, 529–547 (2014).

[b12] Mauck K. E., De Moraes C. M. & Mescher M. C. Infection of host plants by Cucumber mosaic virus increases the susceptibility of Myzus persicae aphids to the parasitoid Aphidius colemani. Sci. Rep. 5, 10963 doi: 10.1038/srep10963 (2015). 26043237 PMC4455285

[b13] Mauck K. E., Smyers E., De Moraes C. M. & Mescher M. C. Virus infection influences host plant interactions with non‐vector herbivores and predators. Funct. Ecol. 29, 662–673 (2015).

[b14] Poelman E. H., Broekgaarden C., Van Loon J. J. & Dicke M. Early season herbivore differentially affects plant defence responses to subsequently colonizing herbivores and their abundance in the field. Mol. Ecol. 17, 3352–3365 (2008). 18565114 10.1111/j.1365-294X.2008.03838.x

[b15] Karban R., Adamchak R. & Schnathorst W. C. Induced resistance and interspecific competition between spider mites and a vascular wilt fungus. Science 235, 678–680 (1987). 17833628 10.1126/science.235.4789.678

[b16] Kessler A. & Baldwin I. T. Defensive function of herbivore-induced plant volatile emissions in nature. Science 291, 2141–2144 (2001). 11251117 10.1126/science.291.5511.2141

[b17] Stephanie K., Andreas K. & Teja T. Interactions between the rust fungus Puccinia punctiformis and ectophagous and endophagous insects on creeping thistle. J. Appl. Ecol. 38, 548–556 (2001).

[b18] Dicke M. & Baldwin I. T. The evolutionary context for herbivore-induced plant volatiles: beyond the ‘cry for help’. Trends Plant. Sci. 15, 167–175 (2010). 20047849 10.1016/j.tplants.2009.12.002

[b19] Rostás M., Ton J., Mauch-Mani B. & Turlings T. C. Fungal infection reduces herbivore-induced plant volatiles of maize but does not affect naive parasitoids. J. Chem. Ecol. 32, 1897–1909 (2006). 16902818 10.1007/s10886-006-9147-3

[b20] Omacini M., Chaneton E. J., Ghersa C. M. & Muller C. B. Symbiotic fungal endophytes control insect host-parasite interaction webs. Nature 409, 78–81 (2001). 11343116 10.1038/35051070

[b21] Kanno H. & Fujita Y. Induced systemic resistance to rice blast fungus in rice plants infested by white‐backed planthopper. Entomol. Exp. Appl. 107, 155–158 (2003).

[b22] Kanno H., Satoh M., Kimura T. & Fujita Y. Some aspects of induced resistance to rice blast fungus, Magnaporthe grisea, in rice plant infested by white-backed planthopper, Sogatella furcifera. Appl. Entomol. Zool. 40, 91–97 (2005).

[b23] Satoh M., Nakajima T. & Kanno H. Induced resistance to rice blast disease in rice plants infested with the white-backed planthopper, Sogatella furcifera, in a paddy field. Jpn. J. Appl. Entomol. Z. 49, 105–111 (2005).

[b24] Simon M. & Hilker M. Herbivores and pathogens on willow: do they affect each other? Agr. Forest Entomol. 5, 275–284 (2003).

[b25] Gomi K., Satoh M., Ozawa R., Shinonaga Y., Sanada S., Sasaki K., Matsumura M., Ohashi Y., Kanno H., Akimitsu K. & Takabayashi J. Role of hydroperoxide lyase in white‐backed planthopper (Sogatella furcifera Horváth)‐induced resistance to bacterial blight in rice, Oryza sativa L. Plant J. 61, 46–57 (2010). 19891707 10.1111/j.1365-313X.2009.04031.x

[b26] McIntyre J., Dodds J. & Hare J. Effects of localized infections of Nicotiana tabacum by tobacco mosaic virus on systematic resistance against diverse pathogens and an insect. Phytopathology 71, 297–301 (1981).

[b27] Bronner R., Westphal E. & Dreger F. Pathogenesis-related proteins in Solanum dulcamara L. resistant to the gall mite Aceria cladophthirus (Nalepa)(syn Eriophyes cladophthirus Nal.). Physiol. Mol. Plant P. 38, 93–104 (1991).

[b28] Simon M. & Hilker M. Does rust infection of willow affect feeding and oviposition behavior of willow leaf beetles? J.Insect Behav. 18, 115–129 (2005).

[b29] Sobhy I. S., Erb M., Lou Y. & Turlings T. C. The prospect of applying chemical elicitors and plant strengtheners to enhance the biological control of crop pests. Philos. T. R. Soc. B. 369, 20120283, doi: 10.1098/rstb.2012.0283 (2014). PMC3928887 24535390

[b30] Tack A. J., Gripenberg S. & Roslin T. Cross‐kingdom interactions matter: fungal‐mediated interactions structure an insect community on oak. Ecol. Lett. 15, 177–185 (2012). 22221681 10.1111/j.1461-0248.2011.01724.x

[b31] Drakulic J., Caulfield J., Woodcock C., Jones S. P., Linforth R. & Bruce T. J. 1. & Ray, R. V. Sharing a Host Plant (Wheat [Triticum aestivum]) Increases the Fitness of Fusarium graminearum and the Severity of Fusarium Head Blight but Reduces the Fitness of Grain Aphids (Sitobion avenae). Appl. Environ. Microbiol. 81, 3492–3501 (2015). 25769834 10.1128/AEM.00226-15 PMC4407206

[b32] Stout M. J., Thaler J. S. & Thomma B. P. Plant-mediated interactions between pathogenic microorganisms and herbivorous arthropods. Annu. Rev. Entomol. 51, 663–689 (2006). 16332227 10.1146/annurev.ento.51.110104.151117

[b33] Hattori M. Probing behavior of the brown planthopper, Nilaparvata lugens Stal (Homoptera: Delphacidae) on a non-host barnyard grass, and resistant and susceptible varieties of rice. Appl. Entomol. Zool. 36, 83–89 (2001).

[b34] Seo B. Y., Kwon Y.-H., Jung J. K. & Kim G.-H. Electrical penetration graphic waveforms in relation to the actual positions of the stylet tips of Nilaparvata lugens in rice tissue. J. Asia-Pac.Entomol. 12, 89–95 (2009).

[b35] Ghaffar M. B. A., Pritchard J. & Ford-Lloyd B. Brown planthopper (N. lugens Stal) feeding behaviour on rice germplasm as an indicator of resistance. Plos. One. 6, e22137; doi: 10.1371/journal.pone.0022137 (2011). 21779386 PMC3136512

[b36] Schröder F. Induced chemical defense in plants. Angew. Chem. Int. Edit. 37, 1213–1216 (1998). 10.1002/(SICI)1521-3773(19980518)37:9<1213::AID-ANIE1213>3.0.CO;2-0 29711238

[b37] Cardoza Y. J. & Tumlinson J. H. Compatible and incompatible Xanthomonas infections differentially affect herbivore-induced volatile emission by pepper plants. J. Chem. Ecol. 32, 1755–1768 (2006). 16900430 10.1007/s10886-006-9107-y

[b38] Kruess A. Indirect interaction between a fungal plant pathogen and a herbivorous beetle of the weed Cirsium arvense. Oecologia 130, 563–569 (2002). 10.1007/s00442-001-0829-9 28547258

[b39] Rostás M. & Hilker M. Indirect interactions between a phytopathogenic and an entomopathogenic fungus. Sci. Nat. 90, 63–67 (2003). 10.1007/s00114-002-0395-y 12590299

[b40] Stout M., Fidantsef A., Duffey S. & Bostock R. Signal interactions in pathogen and insect attack: systemic plant-mediated interactions between pathogens and herbivores of the tomato, Lycopersicon esculentum. Physiol. Mol. Plant. P. 54, 115–130 (1999).

[b41] Robacker D., Warfield W. & Flath R. A four-component attractant for the Mexican fruit fly, Anastrepha ludens (Diptera: Tephritidae), from host fruit. J. Chem. Ecol. 18, 1239–1254 (1992). 24254162 10.1007/BF00980077

[b42] Bruce T. J., Wadhams L. J. & Woodcock C. M. Insect host location: a volatile situation. Trends. Plant. Sci. 10, 269–274 (2005). 15949760 10.1016/j.tplants.2005.04.003

[b43] Beyaert I., Wäschkea N., Scholz A., Varama M., Reinecke A. & Hilker M. Relevance of resource-indicating key volatiles and habitat odour for insect orientation. Anim. Behav. 79, 1077–1086 (2010).

[b44] Bruce T. J. & Pickett J. A. Perception of plant volatile blends by herbivorous insects–finding the right mix. Phytochemistry 72, 1605–1611 (2011). 21596403 10.1016/j.phytochem.2011.04.011

[b45] Tasin M., Knudsen G. K. & Pertot I. Smelling a diseased host: grapevine moth responses to healthy and fungus-infected grapes. Anim. Behav. 83, 555–562 (2012).

[b46] Cardoza Y. J., Teal P. E. & Tumlinson J. H. Effect of peanut plant fungal infection on oviposition preference by Spodoptera exigua and on host-searching behavior by Cotesia marginiventris. Environ. Entomol. 32, 970–976 (2003).

[b47] Dötterl S., Jürgens A., Wolfe L. & Biere A. Disease status and population origin effects on floral scent: potential consequences for oviposition and fruit predation in a complex interaction between a plant, fungus, and noctuid moth. J. Chem. Ecol. 35, 307–319 (2009). 19241105 10.1007/s10886-009-9601-0

[b48] Rizvi S. Z., Raman A., Wheatley W., Cook G. & Nicol H. Influence of Botrytis cinerea (Helotiales: Sclerotiniaceae) infected leaves of Vitis vinifera (Vitales: Vitaceae) on the preference of Epiphyas postvittana (Lepidoptera: Tortricidae). Austral. J. Entomol. 54, 60–70 (2015).

[b49] Niinemets Ü., Kännaste A. & Copolovici L. Quantitative patterns between plant volatile emissions induced by biotic stresses and the degree of damage. Front. Plant. Sci. 4, 229–237 (2013). 10.3389/fpls.2013.00262 PMC3719043 23888161

[b50] McLeod G., Gries R., von Reuss S. H., Rahe J. E., McIntosh R., König W. A. & Gries G. The pathogen causing Dutch elm disease makes host trees attract insect vectors. Proc. Biol. Sci. 272, 2499–2503 (2005). 16271975 10.1098/rspb.2005.3202 PMC1599782

[b51] Mayer C. J., Vilcinskas A. & Gross J. Phytopathogen lures its insect vector by altering host plant odor. J. Chem. Ecol. 34, 1045–1049 (2008). 18600377 10.1007/s10886-008-9516-1

[b52] Mauck K. E., De Moraes C. M. & Mescher M. C. Deceptive chemical signals induced by a plant virus attract insect vectors to inferior hosts. P. Natl. Acad. Sci. USA 107, 3600–3605 (2010). 10.1073/pnas.0907191107 PMC2840436 20133719

[b53] Fereres A. & Moreno A. Behavioural aspects influencing plant virus transmission by homopteran insects. Virus. Res. 141, 158–168 (2009). 19152819 10.1016/j.virusres.2008.10.020

[b54] Bosque-Pérez N. A. & Eigenbrode S. D. The influence of virus-induced changes in plants on aphid vectors: insights from luteovirus pathosystems. Virus. Res. 159, 201–205 (2011). 21549769 10.1016/j.virusres.2011.04.020

[b55] van Molken T., de Caluwe H., Hordijk C. A., Leon-Reyes A., Snoeren T. A., van Dam N. M. & Stuefer J. F. Virus infection decreases the attractiveness of white clover plants for a non-vectoring herbivore. Oecologia 170, 433–444 (2012). 22526939 10.1007/s00442-012-2322-z PMC3439618

[b56] Xue K., Deng S., Wang R., Yan F. & Xu C. Leaf surface factors of transgenic Bt cotton associated with the feeding behaviors of cotton aphids: A case study on non-target effects. Sci. China. Ser. C. 51, 145–156 (2008). 10.1007/s11427-008-0028-6 18239893

[b57] Khan Z. & Saxena R. Effect of steam distillate extracts of resistant and susceptible rice cultivars on behavior of Sogatella furcifera (Homoptera: Delphacidae). J. Chem. Ecol. 79, 928–935 (1986).

[b58] Lou Y.-G. & Cheng J.-A. Role of rice volatiles in the foraging behaviour of the predator Cyrtorhinus lividipennis for the rice brown planthopper Nilaparvata lugens. Biocontrol. 48, 73–86 (2003).

[b59] Sun X., Zhou W., Liu H., Zhang A., Ai C.-R., Zhou S.-S., Zhou C.-X. & Wang M.-Q. Transgenic Bt rice does not challenge host preference of the target pest of rice leaffolder, Cnaphalocrocis medinalis (Lepidoptera: Pyralidae). Plos. One. 8, e79032 doi: 10.1371/journal.pone.0079032 (2013). 24244410 PMC3823965

[b60] Lou Y.-G., Du M.-H., Turlings T. C., Cheng J.-A. & Shan W.-F. Exogenous application of jasmonic acid induces volatile emissions in rice and enhances parasitism of Nilaparvata lugens eggs by theParasitoid Anagrus nilaparvatae. J. Chem. Ecol. 31, 1985–2002 (2005). 16132208 10.1007/s10886-005-6072-9

