# Supplementary material for: Temporal interactions of plant - insect - predator after infection of bacterial pathogen on rice plants
Source: Sci Rep. 2016 May 17;6:26043. doi: 10.1038/srep26043 (PMC4868983; doi:10.1038/srep26043)
Supplement: Supplementary Information [file srep26043-s1.doc]

**Supporting Information**

**Temporal interactions** **of plant -** **insect - predator after** **infection of** **bacterial pathogen on rice plants**

Ze Sun^1#^, Zhuang Liu^1#^, Wen Zhou^1^, Huanan Jin^1^, Hao Liu^1^, Aiming Zhou^1^, Aijun Zhang^2^, Man-Qun Wang^1^*

1. Hubei Insect Resources Utilization and Sustainable Pest Management Key Laboratory, College of Plant Science and Technology, Huazhong Agricultural University, Wuhan 430070, P. R. China

2. Invasive Insect Biocontrol and Behavior Laboratory, BARC-West, USDA-ARS, Beltsville, MD 20705-2350

*Corresponding author. Address: College of Plant Science and Technology, Huazhong Agricultural University, Wuhan 430070, P.R. China. Tel.: (0086) 13627126839 Fax: (0086) -27-87280920. E-mail: mqwang@mail.hzau.edu.cn (M.-Q Wang)

# These authors contribute equally to this work

**Table S1 Comparison of the probing behaviors of BPH on the rice lines in EPG recordings (0-12h)**

| **0-12h** | **Time to 1st N4a(s)** | **Total duration of N4a (s)** | **Total duration of N4b (s)** | **Total duration of N5 (s)** | **Total duration of np (s)** | **Total duration of ph (s)** | **Total number of ph** |
| --- | --- | --- | --- | --- | --- | --- | --- |
| Health rice(n=13) | 2140.62±345.24 | 4804.77±588.24 | 32734.46±1312.61 | 775.23±194.66 | 1559.85±591.93 | 3301.77±794.56 | 7.69±2.00 |
| 1DPI(n=12) | 3796.25±1315.75 | 6471.75±715.73 | 31611.00±1055.95 | 256.50±109.13 | 1194.08±391.07 | 3645.67±659.24 | 9.92±1.22 |
| 2DPI(n=15) | 3472.47±1767.61 | 6803.33±926.02 | 30011.27±2229.66 * | 486.20±172.97 | 2126.67±1236.43 | 3732.73±953.13 | 11.13±1.82 |
| 3DPI(n=12) | 6852.83±2566.05 | 6439.63±1274.99 | 23085.21±2478.86 ** | 1110.25±506.03 | 6724.67±2048.62 * | 5821.75±836.51 | 16.67±2.45 ** |
| 4DPI(n=14) | 1348.71±170.93 | 6537.39±1328.08 | 27728.96±3022.26 | 338.79±87.56 | 2512.29±1206.77 | 3774.21±664.03 | 11.14±1.80 |
| 5DPI(n=15) | 2616.90±872.94 | 6935.80±1520.77 | 27635.70±3165.59 | 1290.40±623.58 | 2105.10±891.40 | 5225.90±1196.94 | 14.60±2.06 * |
| 6DPI(n=9) | 4314.33±1934.43 | 8585.33±2060.82 | 24944.67±3954.79 | 2055.78±1149.06 | 2625.00±1157.71 | 4957.78±1083.80 | 14.67±2.46 * |
| 10d healthy rice(n=10) | 3819.40±1126.00 | 9013.00±1517.30 | 25912.00±2749.61 | 859.20±292.10 | 2491.70±847.98 | 4895.80±1000.62 | 15.00±3.58 |
| 10DPI(n=10) | 2719.90±1203.27 | 7303.00±1048.01 | 29529.80±2062.48 | 881.10±553.69 | 2115.50±816.03 | 3353.70±816.50 | 10.20±1.83 |
| 15d healthy rice(n=12) | 3908.50±1124.93 | 7815.92±1028.27 | 29196.83±1909.21 | 1273.42±436.69 | 2119.33±571.94 | 3244.25±515.80 | 10.25±0.87 |
| 15DPI(n=17) | 3421.41±1098.72 | 6867.32±943.03 | 29213.38±2233.28 | 485.00±191.59 | 2033.06±772.30 | 4586.24±1310.90 | 11.35±2.59 |

Note: Mean±SE followed by asterisks show significant differences from the control group (*p<0.05; **p<0.01)

**Table S2 Volatiles from healthy rice and rice infected by *XOO* (PI) in 5d, 10d and 15d**

| **Compound** | **Healthy rice** | **5DPI** | **10DPI** | **15DPI** |
| --- | --- | --- | --- | --- |
| Toluene | 15.90±2.54 | 21.42±0.56 | 25.96±1.38 | 15.26±8.12 |
| Hexanal | ND | 40.02±5.31 | 44.58±6.34 | 6.59±2.18 |
| Heptane, 2,4-dimethyl- | 66.57±14.27 | 100.12±13.96 | 142.63±44.94 | 25.94±11.56 |
| 2-Hexen-1-ol | 42.94±10.24 | 81.54±14.90 | 69.31±3.66 | ND |
| 1-Hexanol | 35.80±2.43 | 144.71±29.28 | 91.68±2.90 | ND |
| Nonane | 36.16±16.74 | ND | ND | ND |
| Tricyclene | 6.47±0.51 | 24.84±1.71 | 13.03±0.80 | 7.06±3.25 |
| α-Pinene | 243.09±29.72 | 2437.65±137.96 | 1095.67±17.13 | 96.75±34.01 |
| Camphene | 179.24±7.99 | 935.58±62.32 | 557.71±13.64 | 103.39±49.40 |
| β-Pinene | 81.09±46.73 | 117.80±20.11 | 61.13±10.85 | 8.85±0.68 |
| 5-Hepten-2-one, 6-methyl- | 6.94±2.40 | 17.40±9.45 | 19.79±5.92 | 5.75±0.91 |
| Benzene, 1-ethyl,-methyl- | 16.31±0.99 | 20.67±2.25 | 30.27±2.62 | ND |
| n-Octanal | 17.75±0.43 | 25.76±10.56 | 26.15±8.85 | 8.90±0.99 |
| Cymene | ND | ND | 181.83±24.27 | 51.97±8.47 |
| Limonene | 51.80±2.76 | 73.65±14.61 | 104.38±6.06 | 20.51±5.67 |
| β-Phellandrene | 52.19±10.87 | 203.96±55.06 | 140.93±45.99 | 10.36±2.93 |
| Undecane | ND | ND | ND | 11.66±0.63 |
| hydrocarbon-1 | ND | ND | 22.05±2.42 | ND |
| n-Nonanal | 79.03±7.11 | 110.15±43.00 | 92.33±33.77 | 31.62±3.71 |
| Camphor | 38.99±6.46 | 35.86±9.88 | 57.32±8.83 | 21.30±4.45 |
| 1-Nonanol | 26.56±1.23 | 49.42±21.18 | 28.03±5.88 | ND |
| Benzo[c]thiophene | 85.39±4.84 | 58.52±9.88 | 91.76±8.09 | 38.50±8.66 |
| n-Decanal | 102.82±11.99 | 145.50±70.01 | 132.02±41.40 | 55.35±2.66 |
| hydrocarbon-2 | ND | ND | 23.03±0.77 | ND |
| C10H12O (dimethyl-acetophenone, or ethyl acetophenone) | ND | ND | 224.07±37.43 | ND |
| Tridecane | 39.34±3.83 | 38.53±1.71 | 151.72±104.91 | 22.81±4.39 |
| Tridecane, 2-methyl- | 16.18±0.55 | ND | 15.11±3.50 | ND |
| Tridecane, 3-methyl- | 8.89±1.91 | ND | 12.50±1.38 | ND |
| Dodecane, 2,6,10-trimethyl- | 26.74±2.54 | 34.42±1.77 | 33.33±4.81 | 16.02±5.45 |
| Tetradecane | 149.25±14.75 | 153.37±4.33 | 181.82±25.73 | 61.84±15.03 |
| Longifolene | 91.62±7.39 | 111.60±25.44 | 119.74±26.29 | 31.69±4.52 |
| α-Cedrene | 106.18±8.58 | 93.61±15.36 | 101.68±26.92 | 34.76±6.54 |
| Tetradecane, 2-methyl- | 58.62±4.62 | ND | 67.40±6.56 | 25.16±5.83 |
| Tetradecane, 3-methyl- | 16.22±1.34 | ND | ND | 6.16±3.12 |
| Pentadecane | 253.21±20.99 | 248.48±11.86 | 302.24±16.89 | 100.39±25.58 |
| hydrocarbon-3 | 27.93±5.54 | 25.39±1.00 | ND | 10.10±3.13 |
| Pentadecane, 2-methyl- | 64.93±6.05 | 61.25±0.80 | 63.23±13.94 | 25.45±7.52 |
| Pentadecane, 3-methyl- | 28.77±3.58 | 27.30±1.83 | 64.66±18.74 | 9.81±2.63 |
| Hexadecane | 313.84±23.40 | 258.38±0.65 | 383.07±6.43 | 131.21±34.89 |
| Cedrol | 58.51±6.96 | 41.38±4.62 | 68.19±3.10 | 22.37±4.10 |
| Pentadecane, 2,6,10-trimethyl- | 122.63±5.78 | 102.68±4.86 | 140.39±4.93 | 45.45±13.08 |
| hydrocarbon-4 | 31.71±1.51 | 24.40±0.78 | 39.52±2.96 | 10.86±3.03 |
| Heptadecane | 143.66±11.67 | 155.18±39.18 | 174.55±10.73 | 58.76±15.26 |
| Pentadecane, 2,6,10,14-tetramethyl- | 97.14±5.63 | ND | 110.02±2.47 | 37.98±11.56 |
| Octadecane | 33.02±5.18 | 22.31±1.15 | 46.77±4.70 | 28.64±10.09 |
| Hexadecane, 2,6,10,14-tetramethyl- | 59.63±9.93 | 49.24±1.79 | 80.86±3.13 | 27.97±6.88 |

**Note: Mean amounts (% of IS peak area ± SEM, n = 2-4) of volatiles emitted from healthy rice and rice infected by *Xoo*(PI) in 5d,10d and 15d. IS: internal standard. ND: not detected.**
